# New Insights into Health Conditions Related to Malfunctions in Clock Genes

**DOI:** 10.3390/biom14101282

**Published:** 2024-10-11

**Authors:** Kaja Majewska, Mikołaj Seremak, Katarzyna Podhorodecka, Maria Derkaczew, Bartosz Kędziora, Paulina Boniecka, Kamila Zglejc-Waszak, Agnieszka Korytko, Małgorzata Pawłowicz, Joanna Wojtkiewicz

**Affiliations:** 1 Warmian-Masurian Cancer Center of the Ministry of the Interior and Administration Hospital, 10-228 Olsztyn, Poland; kajamaj11@gmail.com; 2 Students’ Scientific Club of Pathophysiology, Department of Human Physiology and Pathophysiology, Faculty of Medicine, Collegium Medicum, University of Warmia and Mazury in Olsztyn, 10-082 Olsztyn, Poland; mikolaj.seremak@gmail.com (M.S.); k.podhorodecka1@gmail.com (K.P.); m.derkaczew@gmail.com (M.D.); bartosz.kedziora97@gmail.com (B.K.); paubon3434@gmail.com (P.B.); 3 Regional Specialist Hospital, 10-561 Olsztyn, Poland; 4 University Teaching Hospital, 10-082 Olsztyn, Poland; 5 Department of Internal Medicine, Hospital of the Ministry of Interior, 25-375 Kielce, Poland; 6 Department of Anatomy, Faculty of Medicine, Collegium Medicum, University of Warmia and Mazury in Olsztyn, 10-082 Olsztyn, Poland; 7 Department of Human Physiology and Pathophysiology, Faculty of Medicine, Collegium Medicum, University of Warmia and Mazury in Olsztyn, 10-082 Olsztyn, Poland; agusiakorytko@gmail.com; 8 Department of Pediatric Neurogenetics and Rare Diseases, Prof. Dr. Stanislaw Popowski Regional Specialized Children’s Hospital, 10-561 Olsztyn, Poland; malgorzata.pawlowicz@uwm.edu.pl; 9 Department of Clinical Pediatrics, Faculty of Medicine, Collegium Medicum, University of Warmia and Mazury in Olsztyn, 10-719 Olsztyn, Poland

**Keywords:** circadian rhythm, chronotype, sleep quality, clock genes, polymorphism

## Abstract

Chronotypes play a crucial role in regulating sleep–wake cycles and overall health. The aim of this study was to investigate chronotype, sleep quality, polymorphisms of clock genes and the level of leptin in serum. We used standardized questionnaires to assess chronotype and sleep quality. Genetic analysis was performed to determine the selected clock gene polymorphism. Serum leptin level was measured by the Elisa method. The results showed that serum leptin concentration was elevated in women, as well as in men who had a high waist-to-hip ratio (WHR) and body mass index (BMI). The evidence indicated that younger students (<22 years old) were most likely to experience poor sleep quality. Nevertheless, our multivariate analysis revealed that young age and a morning-oriented chronotype were associated with better sleep quality. We noted that clock gene polymorphisms were present in 28.6% of the participants. Moreover, polymorphisms of PER1 c.2247C>T (rs2735611) and PER2 c.-12C>G (rs2304672) genes were associated with serum leptin level and chronotype, respectively. These findings provide insights into the relationships between chronotype, sleep quality, clock gene polymorphisms and obesity risk in biomedical students. Understanding these factors can contribute to better sleep management and potential interventions to improve health outcomes in humans.

## 1. Introduction

The circadian rhythm is a complex system that allows for the maintenance of homeostasis in basic adaptive behaviors such as sleep–wake cycles, feeding and procreation [1]. In most individuals, the circadian rhythm typically spans slightly more than 24 h, requiring environmental cues, such as light, to synchronize with the 24 h day–night cycle [2]. The areas responsible for organizing the circadian rhythm in mammals are localized in the suprachiasmatic nucleus in the hypothalamus [3]. The most important factor responsible for synchronizing the circadian clock is exposure to daylight or darkness [4]. Thus, circadian clock disruptions are common in blind people [5].

Nevertheless, humans have individualized sleep time preferences called chronotypes (CTs) that are based on genetics, development and external influences. There are three defined categories of chronotype: morning types (M-types), evening types (E-types) and neither type (N-type) [6,7]. CT refers to the individual differences in sleep–wake patterns, diurnal preferences and alertness in the morning and evening. M-types (usually known as “early larks”) are early risers, perform best mentally and physically in the morning hours and go to bed early in the evening. E-types (commonly called “night owls”) stay up late at night, rise at a later time in the morning and perform best mentally and physically in the late afternoon or evening [8,9,10,11]. CT changes during life, and we can find more early birds among children and the elderly. Morning or evening preference alters in a characteristic manner: children are usually morning types, adolescents tend towards eveningness, with its maximal values around the age of 20, and then the chronotype gradually changes to morning type in older people [12,13,14]. It is worth noting that circadian tendencies are also linked to an individual’s sex. Before the age of 45, men are more likely to prefer an evening lifestyle than women. This relationship changes completely after the age of 45, when men turn out to be morning types more often. Nevertheless, the social role of men is currently growing. Hence, in a few years we may not observe similar differences.

There are many theories to explain this phenomenon, and one of them is molecular aging. Moreover, some findings suggest the activation of previously dormant genes in older people’s genomes. Aging causes many changes in metabolism, such as lowered levels of melatonin and cortisol, and a decrease in core body temperature. All of these processes contribute to shifts in circadian rhythm [14,15]. We assume that changes in chronotype are related to environmental factors but also to the aging process and the increased frequency of neurodegenerative diseases in older people.

In 1984, the period gene was discovered in Drosophila, which is responsible for the regulation of daily biological rhythms through the oscillating expression of the PERIOD protein. The PERIOD protein accumulates in cells at night and then it degrades during the day [16]. We know that the circadian clock mechanism is made up of two interlocking, regulatory feedback loops. The first one includes the clock and Bmal1 genes, whose products initiate transcription of the target genes Period (PER1, PER2, PER3) and Cryptochrome (Cry1, Cry2). The second regulatory loop is also induced by CLOCK:BMAL1 heterodimers, but in this case, it activates transcription of the Rev-erbα and Rorα genes [17,18,19]. Every day, the protein PER accumulates to a certain concentration upon which it enters into the nucleus together with protein CRY. This complex inhibits expression of the PER and CRY genes. After the degradation of the inhibitor complex, the suppression is relieved and a new circadian cycle starts [20]. The generation of the 24 h molecular clock is governed by post-translational modifications such as phosphorylation and ubiquitination. Casein kinase 1 epsilon and Casein kinase 1 delta (CK1ε and CK1δ) are crucial factors that regulate the core circadian protein turnover in mammals [17]. Every tissue in our body has a circadian clock that regulates the synchronization of these loops with environmental factors (such as light and temperature). Nevertheless, studies indicated the influence of light and the circadian system on rhythmic brain function. Furthermore, light at night and disrupted circadian rhythms alter physiology and behavior.

Moreover, perturbations in the circadian clock can promote cardiovascular diseases, metabolic syndrome, obesity and cancer [5]. People who work at night and change time zones are particularly at risk of developing cancer. The study that identified this risk included nurses and drivers working night shifts. Studies have indicated an increased risk of developing colorectal, breast, endometrial and rectal cancer, as well as prostate cancer [5]. However, further studies are necessary to clarify this phenomenon.

The goals of our study were (1) to determine the type of chronotype (definitely evening/moderately evening/intermediate/moderately morning/definitely morning) in the study group based on the standardized Horne–Östberg questionnaire, (2) to evaluate the interrelationship between the established type of chronotype and the sleep quality index (PSQI) calculated on the basis of the standardized Pittsburgh questionnaire in the study group, (3) to analyse of serum leptin levels and selected polymorphisms of the clock genes, i.e., PER1 c.2247C>T (rs2735611), PER2 c.-12C>G (rs2304672), PER3 c.983T>G (rs10462020) and (4) to determine the genotype/chronotype/sleep quality disorder/leptin level system associated with the highest risk of developing obesity in the study group.

## 2. Materials and Methods

### 2.1. Patient Information

Ethical permission was acquired from the Bioethics Committee of the University of Warmia and Mazury in Olsztyn, no. 70/2018. The patient cohort consisted of 155 patients: 107 women and 48 men. Patients were healthy students of the UWM biomedical faculties (medical, nursing and paramedical faculties). Exclusion criteria were type 2 diabetes or prediabetes, diagnosed and treated quantitative and qualitative sleep disorders, epilepsy, diagnosed and treated mood disorders, psychoses, personality disorders and cancer. Patients who did not respond to these questions were not included in the analysis. We used the Pittsburgh Sleep Quality Index and the Morningness–Eveningness Questionnaire. Furthermore, the standardized Horne–Östberg questionnaire for chronotype assessment in each participant was used at the start of the study. The standardized Pittsburgh questionnaire for the assessment of quantitative and qualitative sleep disorders in each project participant was also used at the start of the study. The Morningness–Eveningness Questionnaire, developed by researchers James A. Horne and Olov Östberg in 1976, was originally used to differentiate between the two extreme diurnal types: morning type and evening type [20]. The Morningness–Eveningness Questionnaire measures the preferred time of rising and bedtime, as well as physical and mental performance and alertness after rising and after various activities. We used the Polish version of the rMEQ, which consists of four items, which seems particularly useful for examining morning/evening preferences in large student samples [21]. Whole blood was collected for further analysis. Each participant in the study had their weight, height, waist circumference and hip circumference measured. Based on these data, body mass index (BMI) and fat distribution index (WHR)—ratio of waist circumference to hip circumference—were calculated. Informed consent was obtained from the study participants prior to study commencement.

### 2.2. gDNA Isolation

gDNA was obtained to create a template for clock gene polymorphism studies. Briefly, the gDNA was isolated from a dry droplet of blood collected from participants on Guthrie blotting papers (Whatman Protein Saver Cards, Sigma-Aldrich, Saint Louis, MO, USA) with the use of a Sherlock AX kit (A&A Biotechnology, Gdańsk, Poland), according to the manufacturer’s protocol. The qualification and quantity of the isolated gDNA were measured on an Infinite 200 mol/L PRO spectrophotometer (TECAN, Männedorf, Switzerland). The gDNA was used for further analysis.

### 2.3. Genotyping of PER1/2/3

Polymerase chain reaction (PCR) was used to replicate the target DNA fragment into thousands of copies under controlled conditions using primers (Table 1). Primers were designed with the web-based software PrimerQuest-design qPCR assays: IDT ((PER1 F: 5′-CTGAAGTGGTTCTGATGACCTTT-3′; R: 5′-TGGACGGTAGGCGTCTG-3′), (PER2 F: 5′-CTTGTGCGTGTGCTTGTTAAT-3′; R: 5′-GGAAATTCCGCGTATCCATTCA-3′) and (PER3 F: 5′-CAGATCCTGTCCACGGC-3′; R: 5′-TTCAAAGTAAGAGGCGAGTGT-3′)). The PCR preceded DNA sequencing. Determination of the selected polymorphisms of the clock genes PER1 c.2247C>T (rs2735611), PER2 c.-12C>G (rs2304672) and PER3 c.983T>G (rs10462020) was performed by DNA sequencing (GENOMED S.A., Warsaw, Poland, Table 2). Sequencing and SNP identification were examined by FinchTV (Geospiza, Inc., Seattle, WA, USA) and aligned by DNASIS v.3.0 (Hitachi Software Engineering Co., Ltd., Tokyo, Japan). Next, the sequences were compared with those deposited in the GenBank database (National Center for Biotechnology Information; BLAST).

### 2.4. Enzyme-Linked Immune-Sorbent Assay (ELISA) of Leptin

We collected blood samples to determine leptin levels at approximately the same time in the morning (8–9 a.m.). The concentrations of leptin protein in serum were determined using a commercial ELISA kit (Human Leptin ELISA Kit, Sigma-Aldrich, Saint Louis, MO, USA) according to the manufacturer’s protocol (available online: https://www.sigmaaldrich.com/PL/pl/technical-documents/protocol/protein-biology/elisa/elisa-protocols, accessed on 11 March 2022).

### 2.5. Statistical Analysis

Quantitative data were reported as median (interquartile range) for non-normally distributed data and as numbers (percentages) for qualitative data. The normal distribution of continuous variables was assessed using the Shapiro–Wilk test. In cases where the data did not follow a normal distribution, the Mann–Whitney U test (also known as Wilcoxon rank sum test) was used, and the results were presented as the median (25–75% interquartile range, IQR). Categorical variables were analyzed using the chi-square (χ2) and Fisher’s exact tests. To identify independent predictors of sleep quality, a multivariate logistic regression model was constructed using maximum likelihood estimation. Only variables that showed statistical significance (*p* < 0.05) were included in the multivariate model. Prior to finalizing the model, a multicollinearity analysis was conducted. The calibration of the prediction model was assessed using the Hosmer–Lemeshow goodness-of-fit test. Effect sizes were reported as odds ratios (OR) with 95% confidence intervals (CIs). The *p*-values were calculated using the Wald distribution approximation. A significance level of α < 0.05 was considered nominal. The data analysis for this study was performed using Statistica version 13 (StatSoft Inc., Tulsa, OK, USA) and R-project (version 4.2.3) with the “gtsummary” package [22].

## 3. Results

This study included healthy students from UWM biomedical faculties, with ages ranging from 19 to 31 years (median 23, IQR 22–25 years), who were enrolled in our study. Out of the 155 students, 107 were female and 48 were male. We did not observe significant differences in age or rMEQ score. Nevertheless, serum leptin concentration was significantly higher in women than in men (*p* < 0.001, Table 3). Leptin is the hormone of hunger and satiety. It is secreted by adipose tissue. Currently, it is believed that its primary function is to signal the level of energy reserves in the hypothalamus in order to optimize food intake and energy expenditure [23]. Leptin levels fluctuate significantly throughout the day. Therefore, we asked the study participants to arrive in the morning and on an empty stomach.

Moreover, while both WHR and BMI were significantly higher in men than in women (*p* < 0.001 and *p* = 0.009, respectively; Table 3), the median WHR (IQR) for women was 0.8 (0.7, 0.8) and for men 0.9 (0.85, 0.9), which, although statistically significant, did not exceed the thresholds for abdominal obesity (WHR > 0.85 for women and >0.9 for men).

Previous studies have shown that estrogens can regulate leptin levels, while other studies have shown that estrogens can regulate metabolism. Hence, our studies showed higher leptin levels in women than in men. Moreover, WHR and BMI were significantly higher in men (*p* < 0.001, *p* = 0.009, respectively; Table 3).

We used cluster analysis and k-means to determine the age cut-off point below which sleep quality deteriorated, yielding a value of 21.8 years. We then split our sample into two groups: those under the age of 22 and those 22 or older. By performing univariate analysis with the new variable, we demonstrated that younger students (<22 years old) were more than twice as likely to have poor sleep quality (OR = 2.63, 95% CI 1.20–6.10, *p* = 0.019). In the univariate analysis, statistically significant differences were observed in age, rMEQ scores and chronotype types (Table 4). 

**Figure 1:**
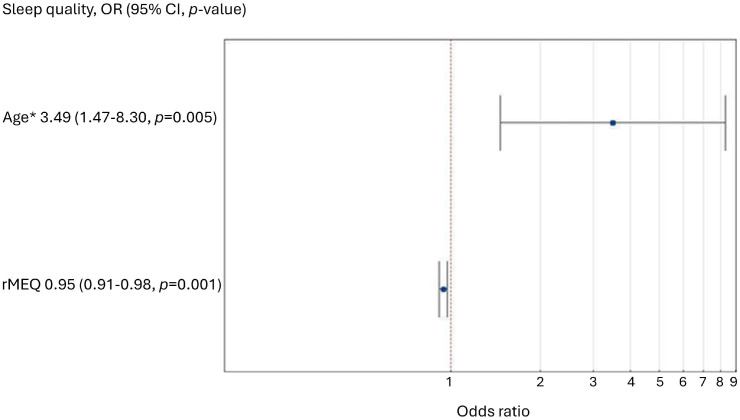
The figure shows risk factors associated with poor sleep quality (* age < 22).

For the multivariate logistic regression model, we chose to include a continuous chronotype variable (rMEQ) instead of its categorical counterpart, as it yielded better fit parameters (lower AIC and BIC values and higher Cox–Snell’s R-squared values). The Hosmer–Lemeshow test for the model gave the result χ2 = 1.69, *p* = 0.790, which indicates a good fit of the logistic regression model. In the multivariate logistic regression model, younger age (<22 years) was associated with an increased likelihood of poor sleep quality (*p* = 0.005). In addition, an increase in rMEQ (a shift toward the morningness chronotype) was linked to a decreased likelihood of poor sleep quality (*p* = 0.001, Figure 1).

Alterations in *PER* gene frequency may affect the circadian phase. Our results indicated that 85.4%, 85.7% and 95.9% of the study population had a predisposed variant allele for the *PER1*, *PER2* and *PER3* genes, respectively (Table 4, Table 5, Table 6, Table 7 and Table 8). The *PER1* non-predisposed variant allele of the gene polymorphism c.2247C>T (rs2735611) was present in 14.6% of people, while the non-predisposed variant allele of *PER2* gene c.-12C>G (rs2304672) was observed in 14.3% of patients. Studies indicated that mutations in *PER1* and -*2* may cause short circadian rhythms in rodents. Next, the *PER3* non-predisposed variant allele of the gene polymorphism c.983T>G (rs10462020) was found in 4.1% of the studied population. Nevertheless, mutations in *PER3* may be associated with nocturnal preferences. Notably, 28.6% of patients had a non-predisposed clock gene allele variant identified in any of the clock genes, indicating a significant prevalence of the non-predisposed variant allele of the clock gene in the cohort. These patients may demonstrate increased vulnerability to sleep–wake disturbances.

We also observed relationships between the selected non-predisposed clock variant allele of genes (*PER1* c.2247C>T rs2735611, *PER2* c.-12C>G rs2304672 and *PER3* c.983T>G rs10462020) and five variables (chronotype, serum leptin levels, BMI, WHR and rMEQ; Table 5, Table 6, Table 7 and Table 8). Thus, the *PER* gene family may regulate metabolism, circadian rhythm and health status. Mutations in clock genes may disrupt leptin levels and contribute to increased BMI and WHR. Therefore, we observe relationship loops between leptin levels, BMI, WHR and mutations in clock genes. The highest level of leptin in the blood occurs between midnight and the early morning hours, while the lowest levels are from mid-morning to midnight. Our study showed that patients with mutations in the *PER1* and *PER2* genes showed an eveningness chronotype. It should be noted that we are dealing with a young group of examined patients. Our studies have shown the lowest leptin levels in patients with a polymorphism in the *PER1* gene, while the highest leptin levels were observed in patients with a polymorphism in the *PER3* gene. Thus, leptin levels may be associated with *PER1/2/3* genes.

Leptin concentrations were significantly lower in the group of patients with the non-predisposed variant of the *PER1* gene allele (*PER1* c.2247C>T (rs2735611) (0.16 ng/mL vs. 0.62 ng/mL, *p* = 0.032; Table 5)). Individuals with the non-predisposed variant of the *PER2* gene allele (*PER2* c.-12C>G (rs2304672)) had significantly lower rMEQ scores, indicating a more evening-oriented chronotype (*p* = 0.05; Table 6). We did not observe any cross-talk between BMI and non-predisposed variants of the *PER1/2/3* gene allele with higher risk (*p* < 0.05, *p* = 0.068, *p* < 0.05, respectively; Table 5, Table 6, Table 7 and Table 8). We did observe a molecular dialog between leptin levels and mutations in clock genes.

## 4. Discussion

In the current study, a sample of healthy college students were examined to determine the association between age, chronotype and sleep quality. Our research identified a number of important relationships and has shed light on the variables affecting this population’s sleep quality. It should be emphasized that our study is focused on students and all information included in our manuscript relates to a specific age group; young adults. Many studies have been conducted on the molecular mechanisms underlying the mammalian circadian rhythm and it has been shown that circadian gene defects can lead to many biological consequences such as metabolic dysfunctions or psychiatric disorders [17,24,25].

Our results have revealed that the level of leptin was higher in females than in men. The hormone leptin, which is mostly released by adipose tissue, is essential for controlling hunger and energy balance [25]. It is possible that differences in body composition and hormonal effects are the reason for higher leptin levels in females. Our results confirm previous results that the level of leptin is gender-specific with females typically having larger amounts [26]. Moreover, we found that men had significantly higher body mass index (BMI) and waist-to-hip ratio (WHR) values compared to women. Importantly, the WHR index has different normal ranges for men and women. According to the WHO guidelines, abdominal obesity is defined as a WHR > 0.85 for women and >0.9 for men. In our study, the median WHR for women was 0.8 (0.7, 0.8) and for men, 0.9 (0.85, 0.9), meaning that, while statistically significant, the difference did not have clinical relevance as both values remained below the WHO cut-offs for abdominal obesity. Nevertheless, increased WHR and BMI are recognized risk factors for a number of illnesses, including sleep disorders and cardiovascular disease [27,28]. Men were found to have higher WHR and BMI values, which may indicate a link between these anthropometric measurements and sleep quality in this sample. The underlying mechanisms relating male students’ body composition and sleep difficulties require further research.

Moreover, we used cluster analysis, k-means and the univariate logistic regression model approach to examine the connection between age and sleep quality. According to our findings, a cut-off age of 21.8 years was associated with a decrease in sleep quality. On the contrary, students under the age of 22 showed a more than two-fold increase in the likelihood of poor sleep quality. These results are consistent with earlier studies showing how young people are more susceptible to sleep disruptions [29]. Early adulthood and adolescence see considerable changes in sleep patterns, including increased sleep debt and delayed sleep–wake schedules [30]. The age-related differences found highlight the importance of managing sleep problems among university students, especially at the beginning of their academic careers.

Moreover, our multivariate logistic regression analysis showed that the odds of having poor sleep quality were reduced when the chronotype shifted toward morningness (higher rMEQ score). Our results and earlier studies [31,32] have revealed that chronotype may have an impact on sleep patterns as well as sleep disorders. People with a more evening-oriented chronotype may have trouble sticking to regular sleep schedules, which can result in sleep disruptions and poorer sleep quality [33,34,35,36]. Understanding different chronotypes can be used to better personalize therapy and instructional approaches to help university students sleep better.

The studies indicate an overall prevalence of clock gene polymorphisms in the cohort. In 28.6% of individuals, we observed a polymorphism in at least one of the studied genes. Our results suggest a significant influence of genetic variation on the regulation of circadian rhythms in the study population. These findings support the notion that clock gene polymorphisms may contribute to inter-individual differences in sleep–wake patterns, chronotype preferences and susceptibility to circadian-related disorders. Our results have indicated the potential for developing personalized interventions to improve sleep and overall health based on an individual’s chronotype and clock gene polymorphisms.

The microbiome plays a crucial role in regulating circadian rhythms and maintaining sleep patterns [37,38]. Moreover, the microbiome stabilizes circadian rhythms in the gut. This phenomenon may be explained by buffering against rapid fluctuations in environmental conditions, thereby stabilizing circadian cycles in the host gut [37,38,39].

Nevertheless, further investigation is required to elucidate the underlying mechanisms of this phenomenon.

The associations between particular clock gene polymorphisms (PER1 c.2247C>T rs2735611, PER2 c.-12C>G rs2304672 and PER3 c.983T>G rs10462020) as well as sleep-related factors were also investigated. Our findings suggested a possible link between clock genes and hormone regulation, as the PER1 c.2247C>T (rs2735611) polymorphism was associated with noticeably reduced leptin concentrations. Similarly, individuals with the PER2 polymorphism c.-12C>G (rs2304672) had a more evening-oriented chronotype as shown by lower rMEQ scores. These results reinforce the understanding of the genetic basis of chronotype and emphasize the function of clock genes in controlling circadian rhythms as well as sleep patterns [33,34,35,36]. However, additional studies with larger samples are needed to confirm these findings and explore potential gene–environment interactions.

This study provides insightful information on the variables that affect students’ sleep quality. The results indicate gender variations in leptin levels and anthropometric measurements, with men having higher WHR and BMI values and women having higher leptin levels. Age was found to be a significant predictor of poor sleep quality, with younger students (under 22) being more susceptible. In addition, chronotype was found to have a significant effect, with morningness orientation being associated with a lower risk of poor sleep quality.

It is extremely important to recognize some of the limitations of our study. First, the cross-section design limits our ability to determine the temporal and causal relationships between the observed connections. Second, the sample size was somewhat small, especially when analyzing clock gene polymorphisms, which would have reduced the statistical power to identify specific relationships. Moreover, the use of self-reported sleep quality ratings and chronotypes in the study creates the potential for errors in remembering as well as reporting. Despite these limitations, our study complements the growing body of research on sleep quality among university students. However, further studies are necessary to explain the cross-talk between sleep quality and genetics.

## 5. Conclusions

Chronotype refers to individual differences in sleep–wake patterns and diurnal preferences, with disruptions in circadian rhythm being associated with various health conditions. Understanding the impact of clock gene polymorphisms on chronotype and sleep quality can provide valuable insights into the risk factors for developing obesity in this population. This study attempts to explore the relationships between chronotype, sleep quality, clock gene polymorphisms and serum leptin levels in biomedical students.

Our findings have shed light on the associations between chronotype, sleep quality and clock gene polymorphisms in biomedical students. The study reveals that younger age and an evening-oriented chronotype are linked to poor sleep quality, while specific clock gene polymorphisms are associated with serum leptin level and chronotype. These findings have implications for sleep management and potential interventions to improve health outcomes, particularly in preventing obesity among biomedical students. Further research is needed to explain this phenomenon.

## Figures and Tables

**Table 1:** Selected circadian genes’ single-nucleotide polymorphisms subject to analysis (National Library of Medicine, National Center of Biotechnology Information).

Gene	Organism	SNP ID	Chromosome	Localization	Alleles	Variation Type
*PER1* c.2247C>T	*Homo sapiens*	rs2736611	17	8144965	C/T	SNV
*PER2* c.-12C>G	*Homo sapiens*	rs2304672	2	238277948	G/C	SNV
*PER3* c.983T>G	*Homo sapiens*	rs10462020	1	7820623	T/G	SNV

Abbreviation: SNP—single-nucleotide polymorphism, SNV—single-nucleotide variation.

**Table 2:** Variant allele of clock genes in the studied population.

Gene (Official Symbol)	Predisposed Allele Variant of the Studied Population	Non-Predisposed Allele Variant of the Studied Population
*PER1*	*PER1* c.2247C	*PER1* c.2247T
*PER2*	*PER2* c.-12C	*PER2* c.-12G
*PER3*	*PER3* c.983T	*PER3* c.983G

**Table 3:** Baseline demographic and clinical characteristics of participants.

Characteristic	n	Women, n = 107	Men, n = 48	*p*-Value ^1^
Age, median (IQR)	148	23 (22, 25)	24 (22, 25)	0.3
rMEQ, median (IQR)	155	44 (37, 53)	42 (38, 49)	0.5
Chronotype, n (%) Morningness Neither Eveningness	155	15 (14%) 45 (42%) 47 (44%)	7 (15%) 19 (40%) 22 (46%)	>0.9
Leptin [ng/mL] Median (IQR)	61	1.21 (0.47, 2.24)	0.15 (0.09, 0.35)	<0.001
BMI (IQR)	154	21.52 (19.9, 23.16)	23.36 (20.38, 24.7)	0.009
WHR (IQR)	69	0.8 (0.7, 0.8)	0.9 (0.85, 0.9)	<0.001

^1^ Wilcoxon rank sum test, Pearson’s chi-squared test.

**Table 4:** Univariable logistic regression analysis evaluating the impact of selected factors on the risk of poor sleep quality.

Characteristic	n	OR ^1^	95%, CI ^1^	*p*-Value
Age	147	0.94	0.82, 1.06	0.3
Age ≥22 <22	147	1.00 2.63	Ref. 1.2, 6.1	0.019
Sex Male	154	0.81	0.41, 1.61	0.6
*PER1* c.2247C>T (rs2735611) polymorphism	48	0.71	0.13, 3.63	0.7
*PER2* c.-12C>G (rs2304672) polymorphism	49	2.75	0.53, 20.7	0.3
*PER3* c.983T>G (rs10462020) polymorphism	49	0.96	0.04, 25.2	>0.9
*PERX* higher risk polymorphism	49	0.94	0.27, 3.31	>0.9
rMEQ	154	0.95	0.92, 0.98	0.001
Chronotype, n (%) Morningness Neither Eveningness	154	1.00 3.51 4.42	Ref. 1.22, 11.7 1.55, 14.7	0.027 0.008
Leptin [ng/mL]	60	1.1	0.89, 1.41	0.4
BMI	153	1.07	0.95, 1.2	0.3
WHR	68	0.83	0.00, 561	>0.9

^1^ OR—odds ratio, CI—confidence interval.

**Table 5:** Effect of the presence of a polymorphism in the *PER1* c.2247C>T (rs2736611) gene on the parameters studied.

Characteristic	n	None, n = 41 ^1^	Polymorphism, n = 7 ^1^	*p*-Value ^2^
Chronotype Morningness Neither Eveningness	48	6 (15%) 19 (46%) 16 (39%)	2 (29%) 2 (29%) 3 (43%)	0.2
Leptin [ng/mL]	39	0.62 (0.23, 1.71)	0.16 (0.13, 0.26)	0.032
BMI	47	21.75 (19.8, 23.7)	23 (20.45, 25.8)	0.4
WHR	47	0.8 (0.7, 0.8)	0.8 (0.75, 0.9)	0.3
rMEX	48	42 (36, 53)	55 (41, 62)	0.2

^1^ n (%); Median (IQR). ^2^ Fisher’s exact test; Wilcoxon rank sum test.

**Table 6:** Effect of presence of a polymorphism in the *PER2* c.-12C>G (rs2304672) gene on the parameters studied.

Characteristic	n	None, n = 42 ^1^	Polymorphism, n = 7 ^1^	*p*-Value ^2^
Chronotype Morningness Neither Eveningness	49	9 (21%) 20 (48%) 13 (31%)	0 (0%) 2 (29%) 5 (71%)	0.13
Leptin [ng/mL]	40	0.51 (0.17, 1.26)	0.43 (0.19, 1.11)	0.9
BMI	48	21.5 (19.8, 23.7)	23.8 (22.25, 24.75)	0.068
WHR	48	0.8 (0.7, 0.8)	0.8 (0.78, 0.85)	0.6
rMEX	49	46 (35, 55)	36 (34, 40)	0.05

^1^ n (%); Median (IQR). ^2^ Fisher’s exact test; Wilcoxon rank sum test.

**Table 7:** Effect of presence of a polymorphism in the *PER3* c.983T>G (rs10462020) gene on the parameters studied.

Characteristic	n	None, n = 47 ^1^	Polymorphism, n = 2 ^1^	*p*-Value ^2^
Chronotype Morningness Neither Eveningness	49	9 (19%) 21 (45%) 17 (36%)	1 (50%) 1 (50%) 0 (0%)	0.13
Leptin [ng/mL]	40	0.51 (0.18, 1.26)	2.2 (1.17, 3.24)	>0.9
BMI	48	22 (19.83, 23.95)	20.25 (19.62, 20.88)	0.3
WHR	48	0.8 (0.7, 0.8)	0.8 (0.8, 0.8)	0.8
rMEX	49	43 (36, 55)	41 (40, 42)	0.7

^1^ n (%); Median (IQR). ^2^ Fisher’s exact test; Wilcoxon rank sum test.

**Table 8:** Effect of the presence of polymorphism with higher risk in any of the genes (*PER1, PER2, PER3*) on the parameters studied.

Characteristic	n	None, n = 35 ^1^	Polymorphism, n = 14 ^1^	*p*-Value ^2^
Chronotype Morningness Neither Eveningness	49	6 (17%) 17 (49%) 12 (34%)	6 (43%) 5 (36%) 3 (21%)	0.7
Leptin [ng/mL]	40	0.57 (0.23, 1.36)	0.26 (0.13, 0.78)	0.3
BMI	48	21.5 (19.65, 23.58)	22.70 (21.2, 25.03)	0.1
WHR	48	0.8 (0.7, 0.8)	0.8 (0.76, 0.88)	0.4
rMEX	49	43 (38, 54)	43 (36, 57)	>0.9

^1^ n (%); Median (IQR). ^2^ Fisher’s exact test; Wilcoxon rank sum test.

## Data Availability

Data are available in the Materials and Methods section as well as the Results section.
